# Ultrasonic agitation reduces the time of calcium hydroxide antimicrobial effect and enhances its penetrability

**DOI:** 10.1007/s10856-021-06607-6

**Published:** 2021-12-07

**Authors:** Flaviana Bombarda de Andrade, Layla Reginna da Silva Munhoz Vasconcelos, Thais Cristina Pereira, Roberto Brandão Garcia, Clóvis Monteiro Bramante, Marco Antônio Hungaro Duarte

**Affiliations:** Department of Operative Dentistry, Endodontics and Dental Materials, Bauru School of Dentistry, University of São Paulo, Bauru, SP Brazil

## Abstract

*Objectives*: The objective of the present work was to evaluate the ultrasonic agitation, time and vehicle (propylene glycol or distilled water) on the antimicrobial potential and penetrability of calcium hydroxide pastes on infected dentin by means of Confocal Laser Scanning Microscopy (CLSM) and microbiological culture (MC). *Materials and methods*: Dentin specimens were infected with *Enterococcus faecalis* using a new contamination protocol of 5 days. The specimens were divided into eight groups and dressed with the pastes for 7 or 15 days: G1) calcium hydroxide (CH) + propylene glycol (prop)/7 days (d), G2) CH + prop/7d + ultrasonic agitation (U), G3) CH + distilled water (dw)/7d, G4) CH + dw/7d + U, G5) CH + prop/15d, G6) CH + prop/15d + U, G7) CH + dw/15d, G8) CH + dw/15d + U. The ultrasonic activation was made for 1 min in both directions with a plain point insert. After medications removal, the images obtained by CLSM showed the viable (green) and dead (red) bacteria with Live and Dead dye. By the MC, the dentinal wall debris obtained by burs were collected for colony counts. For the penetration test, the Rodamine B dye was added to the CH pastes and analyzed by CLSM. *Results:* The 7 and 15-days CH + prop+U pastes performed better antimicrobial efficacy, followed by the CH + dw+U/15d paste. *Conclusions:* All pastes demonstrated better penetration and antimicrobial activity against *E. faecalis* when agitated with ultrasound, even in periods of up to seven days. The propylene glycol vehicle showed better results. *Clinical relevance:* Agitation of the dressing that remains for less time inside the root canal can optimize the decontamination of endodontic treatment.

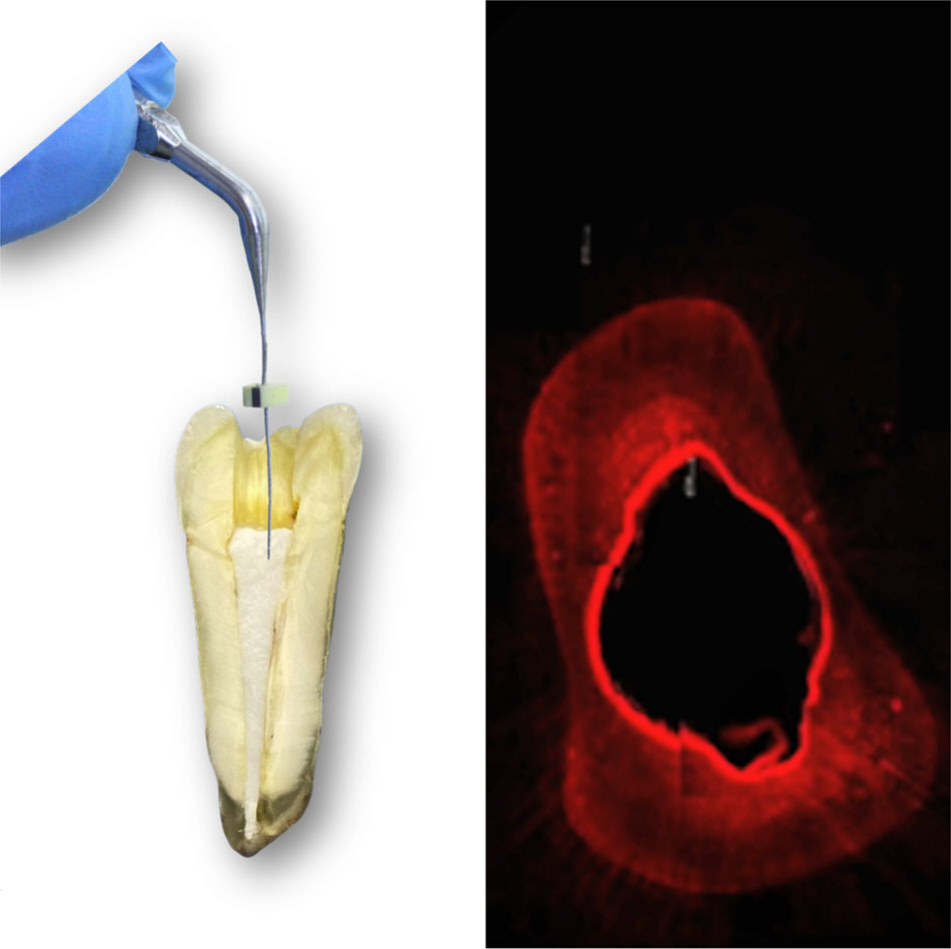

## Introduction

The success of endodontic treatment depends on the reduction or elimination of the intraradicular infection [1–3]. The chemo-mechanical preparation is responsible for the major bacterial elimination, but this procedure is not a guaranty that all the bacteria was eliminated, thus, in the more contaminated cases, the intracanal medication with antimicrobial effect is recommended [4], such as old necrotic cases and retreatments. The most used medication is the calcium hydroxide paste because it presents antibacterial [5] and antiexudative action, and biological induction of mineralized tissue formation, contributing to the repair [6].

The utilized vehicles for calcium hydroxide paste interfere with ionic dissociation, exerting a direct influence to the pH values [7] and biological activities over bacteria and tissues [8]. The vehicle should permit the penetration of the medication through areas that were not touched by the instrumentation and irrigation, therefore, killing the remaining bacteria [9]. Propylene glycol is a colorless liquid that has a light antimicrobial effect, showing beneficial advantages since it provides the intracanal medication to stay for a prolonged time [9–11].

The calcium hydroxide paste is able to eliminate *Enterococcus faecalis* by direct contact [5], although these bacteria can survive until pH 11.5 [12]. However, this microorganism has great capacity to penetrate inside the dentinal tubules [13], and therefore, it is less affected by the medication since it can be found in retreatment cases [14, 15]. Thus, a clinical procedure should be done to improve the penetration of calcium hydroxide paste to be in direct contact with all bacteria. Presently, only the extended time is used to reach this aim. The paste should be inside the root canal for at least 14 days to get a satisfactory result for the elimination of microorganisms [16].

Duarte et al. [17] introduced the ultrasonic agitation of the medication improving the pH and the calcium liberation at the external root surfaces in simulated root resorptions. By means of this physical procedure, this paste can be driven inside the dentinal tubules and then, there is a possibility that it can deeply act on viable bacteria. By means of a recently developed method in vitro [18], it is possible to obtain a model of maximum and homogeneous contamination of teeth to verify the intratubular antimicrobial effect. Also, the confocal laser scanning microscopy (CLSM) is a method that provides the deep visualization of the dentinal tubules to study antimicrobial potential and the penetrability of the pastes [19].

The best penetration and subsequent best antimicrobial effect of calcium hydroxide paste was demonstrated in the study of Arias et al. [20], when using the ultrasonic agitation of the paste with the same efficient contamination protocol in extracted teeth. Therefore, we should evaluate the vehicles and time of using these intracanal medications, with ultrasonic agitation.

The null hypothesis was the ultrasonic agitation does not favor the intratubular antimicrobial action of calcium hydroxide pastes in only seven days. Also, the null hypothesis that propylene glycol and distilled water as paste vehicles penetrate equally inside the tubules was tested.

## Material and methods

Recently extracted bovine teeth (*n* = 168) obtained from a slaughterhouse were utilized, 21 teeth for each experimental group, 10 of them submitted to microbiological culture (MC) and 6 for microbiological CLSM evaluation and 5 for penetrability evaluation. Twenty other teeth were used as positive and negative controls. Specimens number were calculated before the experiment, using G* Power v 3.1 for Mac software (Heinrich Heine, University of Düsseldorf, Germany). The alpha-type error of 0.05 and the beta power of 0.95 were stipulated. Pilot tests were performed on top of the sample calculation, and also, these numbers were based on the experience of our group by the publications of Arias et al. [20] and Pereira et al. [11]. The teeth were sectioned in a cutting machine (Isomet, Buehler, IL, USA) with refrigeration to remove the crown and the 5 apical millimeters (because of the greater anatomical variation), standardizing these dentinal tubes with 12 mm heights. These specimens were instrumented with endodontic files until the diameter of 1.2 mm, 120 K file, (Dentsply/Maillefer, Ballaigues, Vaud, Switzerland). The specimens were submitted to an ultrasonic bath in 1% sodium hypochlorite (Biodinâmica Química e Farmacêutica Ltda., Ibiporã, PR, Brazil), followed by 17% EDTA (Biodinâmica Química e Farmacêutica Ltda., Ibiporã, PR, Brazil) and saline solution for 10 min each to decontaminate. The outer surface of the root canal specimens was coated with nail varnish to prevent seepage of the inoculum. The specimens were sterilized in an autoclave at 121 °C for 24 min inside microtubes with saline solution.

A bacterial reference strain of *Enterococcus faecalis* was acquired (ATCC 29212) and the colonial morphology in Petri dishes and Gram staining confirmed its purity. The microorganism was cultivated in BHI broth (Brain Heart Infusion, Difco, Detroit, Michigan, USA), and transferred through successive subcultures. Dilutions were performed based on the absorbance obtained by the turbidity in a spectrophotometer (SF325NM, Bel Photonics do Brasil Ltda, Osasco, São Paulo, Brazil) and related to the Mc Farland scale.

The prepared specimens were inserted into microtubes containing 1 mL of BHI broth and submitted to an ultrasonic bath for 15 min to promote the better penetration of the broth inside the dentinal tubules. Next, the standardized contamination with *E. faecalis* was performed for 5 days according to Andrade et al. [18], with successive changes of culture media, aseptically, inside a hood and cycles of centrifugation adopted from Ma et al. [19]. After contamination, the specimens were sealed with Coltosol (Coltene, Vigodent do Brasil, Rio de Janeiro, RJ, Brazil) at the apical aperture of the root canal.

Calcium hydroxide powder (Biodinâmica, Londrina, PR, Brazil) was utilized with the vehicles distilled water and propylene glycol. The manipulation of medications was performed in a 2:1 ratio (by weight). The medication groups were distributed as follows: G1) calcium hydroxide paste + propylene glycol/7 days; G2) calcium hydroxide paste + propylene glycol/7 days + ultrasound; G3) calcium hydroxide paste + distilled water/7 days; G4) calcium hydroxide paste + distilled water/7 days + ultrasound; G5) calcium hydroxide paste + propylene glycol/15 days; G6) calcium hydroxide paste + propylene glycol/15 days + ultrasound; G7) calcium hydroxide paste + distilled water/15 days; G8) calcium hydroxide paste + distilled water/15 days + ultrasound. The pastes were inserted in the root canals with #90 K files (Dentsply Maillefer, Ballaigues, Switzerland) with a standardized amount for all specimens. The negative control teeth were not infected. They only received the pastes from each group and the teeth that were used as a positive control received the contamination protocol but were not filled with the medications.

For the four ultrasonic agitation groups, a piezoelectric device was used at a frequency of 30,000 Hz (Emissonic MMO Jardim São Carlos, São Carlos, SP, Brazil). The pastes were also inserted into the specimens with #90 K files, then agitated for 1 min with a sterilized plain point insert Irrisonic E1 (Helse, Santa Rosa de Viterbo, São Paulo, Brazil), 30 s at the mesial-distal direction and 30 s at the buccal-lingual direction, 2 millimeters short of the specimen length.

The medication was maintained inside the specimens for a 7-day period for Groups 1, 2, 3 and 4, and for 15 days in the other groups, including in the negative controls, and stored in microtubes with 1 mL of sterilized distilled water at 37 °C.

### Confocal laser scanning microscopy (CLSM) analysis

After the experimental periods, the intracanal medication of all groups was removed (*n* = 6) with irrigation of sterilized saline solution. The specimens were longitudinally sectioned with a diamond disc (EXTEC, Enfield, USA, 102 mm × 0.3 mm × 12 mm) in the refrigerated cutting machine. The specimens were immersed in 17% EDTA in 24-well plates for 5 min to remove the smear layer produced during the cutting and washed with 500 µL of sterilized saline solution. The control group and pilot studies could prove that 17% EDTA after cutting the teeth did not influence the intratubular contamination since this acid is a weak antimicrobial solution [21].

The specimens were stained with 30 µL of the LIVE/DEAD^®^ BacLight^TM^ Bacterial Viability Kit (Invitrogen Molecular Probes, Eugene, OR, USA) for 20 min. This kit is comprised of green SYTO^®^9 dye and red propidium iodine dye, staining alive and dead bacteria, respectively. The specimens were visualized with the Leica TCS-SPE CLSM (Leica Microsystems GmbH, Mannheim, Germany) obtaining images from the cervical and medium thirds sequentially and divided into superficial and deep regions. The scanning was performed with ×40 magnification at each 1 µm depth and area of 275 µm². The images were acquired through the Leica Application Suite-Advanced Fluorescence software (LAS AF, Leica Mannheim, Germany) and transferred to the Leica LAS AF Lite software to perform the fragmentation of layers of each image. The alive and dead bacteria were quantified through the green and red fluorescence using the bioImagel v2-1 software [22].

### Microbiology culture analysis

The intracanal medication was removed from the ten specimens of each group with saline solution and 0.5% citric acid [23, 24], to neutralize the alkaline pH of the calcium hydroxide. The dentinal tubes were dried with sterilized absorbent paper points (Dentsply Maillefer, Ballaigues, Switzerland). Dentin chips were extracted from the specimens with Largo burs numbers 5 and 6 (Dentsply/Maillefer, Ballaigues, Switzerland) with the aid of an electric motor (VDW, Munich, Germany) with 410 rotations per minute, so as to not promote the heating of the specimen that could interfere with the bacterial growth. One different sterilized bur was used for each specimen. The chips were obtained for 70 s, time standardized in a pilot study, and dispensed immediately in microtubes with BHI broth, positioned below the teeth with a sterilized device. The content of the microtubes were agitated and spread (100 μL) on BHI agar plates. All plates were incubated at 37 °C for 48 h to allow the counting of colony forming units per milliliter (CFU/mL).

The contamination after the intracanal medication of the superficial region (near the canal) was represented by the samples collected with the #5 Largo burs, while the samples of the #6 Largo burs represented the contamination of the deep region of the dentinal mass. Between the use of #5 and #6 bur, 10 mL of saline solution was irrigated to remove the dentine debris which were smeared on to the root canal walls. All procedures involving MC were performed inside a hood, avoiding contamination of the specimens.

### Penetrability of the pastes by CLSM

Twenty other bovine teeth were divided into four groups: Group A) calcium hydroxide + propylene glycol; Group B) calcium hydroxide + propylene glycol + ultrasound; Group C) calcium hydroxide + distilled water; Group D) calcium hydroxide + distilled water + ultrasound. The specimens were prepared in the same manner as the other tests, but without bacterial contamination. The pastes added with rodhamine B dye were inserted into the root canals and remained for 7 days. The teeth were stored in microtubes with 500 microliters of distilled water. After the removal of medications with saline solution and dried with paper points, the specimens were transversally sectioned in the cutting machine at 3 and 5 millimeters from the apical border. These faces were visualized at the Leica TCS-SPE CLSM with ×40 magnification, and the images were acquired through the Leica Application Suite-Advanced Fluorescence software (LAS AF). The images were transferred to the Image J 1.48 v software (National Institutes of Health, USA) to perform the measures of intratubular penetration. The area of the canal and the penetration of the pastes were measured and subtracted with the help of a transparent plastic sheet tagged with some dots.

The statistical analyses of all methodologies were performed after the verification of the normality by the Shapiro–Wilkis and Komorov–Smirnoff tests. The results were not parametric, so, the Kruskal–Wallis followed by the Dunn tests were utilized with 5% of significance.

## Results

### Confocal laser scanning microscopy (CLSM) evaluation

**Fig. 1:**
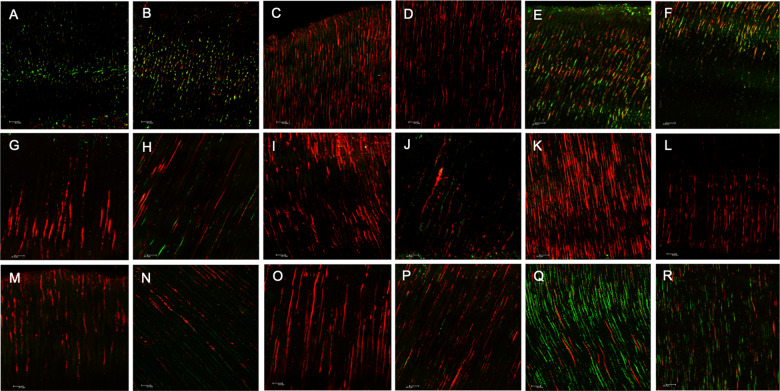
Images of contaminated dentin after using the medications at the superficial (left column) and deep areas (right column) (**A** and **B**). Calcium hydroxide with the vehicle propylene glycol, 7 days (G1). **C** and **D** Calcium hydroxide with the vehicle propylene glycol, 7 days, with ultrasonic agitation (G2). **E** and **F** Calcium hydroxide with the vehicle distilled water, 7 days (G3). **G** and **H** Calcium hydroxide with the vehicle distilled water, 7 days with ultrasonic agitation (G4). **I** and **J** Calcium hydroxide with the vehicle propylene glycol, 15 days (G5). **K** and **L** Calcium hydroxide with the vehicle propylene glycol, 15 days, with ultrasonic agitation (G6). **M** and **N** Calcium hydroxide with the vehicle distilled water, 15 days (G7). **O** and **P** Calcium hydroxide with the vehicle distilled water, 15 days, with ultrasonic agitation (G8). **Q** and **R** Positive control of the bacterial contamination without medication. These images are from the cervical third

The CLSM analysis showed, according to the bacterial viability detected in the images, that Groups G1 (calcium hydroxide + propylene glycol + 7 days), and G3 (CH + distilled water + 7 days) showed the worst results, with statistical differences with the other tested groups (Fig. 1, A-R).

**Fig. 2:**
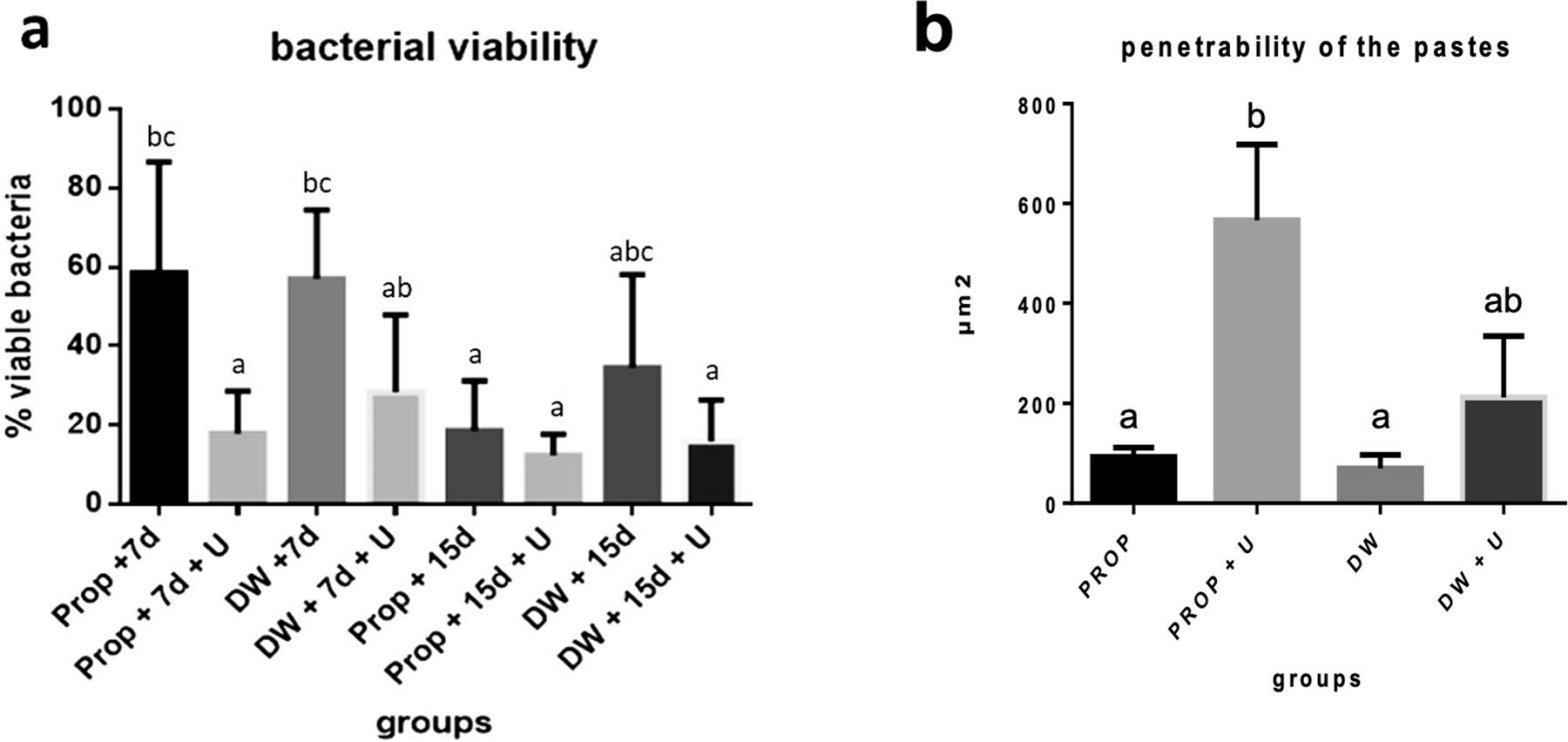
**A** Percentage of alive bacteria at the selected areas (µm^3^), after intracanal medication in the different experimental groups by means of laser confocal scanning microscopy, with or without ultrasound. **B** Medians of penetration areas of the medication of the different tested groups

The groups where the medication remained for 15 days inside the root canals had satisfactory results, presenting less amounts of viable bacteria, without statistical differences between them. The groups where the ultrasonic agitation of medications was employed (G2, G4, G6, and G8) showed great effectiveness to inactivate bacteria, without statistical differences between them, regardless of the time that the pastes remained in the canals. G2 and G4 groups (7 days with ultrasound) did not show statistical differences compare to the G5, G6, G7 and G8 groups (15 days with the pastes), presenting the same effectiveness to eliminate the microorganisms (Fig. 2A).

When the groups were compared in the thirds of the root canals, more effectiveness was observed at the cervical third. In this third, the groups where the paste was agitated with ultrasound maintained for 15 days had the best results, but without statistical differences compared to the groups that received agitation for 7 days and groups without agitation for 15 days (Table 1).

**Table 1:** Values of percentage of viable bacteria inside dentinal tubules after treatment with calcium hydroxide pastes with two different vehicles and two different times, with or without ultrasonic agitation in both cervical and medium thirds of the contaminated roots

Groups	G1	G2	G3	G4	G5	G6	G7	G8	C+
	Prop + 7d	Prop + 7d + U	DW + 7d	DW + 7d + U	Prop + 15d	Prop + 15d + U	DW + 15d	DW + 15d + U	
Cervical third									
Median	64.31^B^	17.82^AB^	58.96^B^	20.21^AB^	13.73^AB^	9.240^A^	19.25^AB^	9.775^A^	69.15
Min–Max	10.64–92.93	5.58–29.91	32.22–80.74	4.18–35.41	3.24–38.63	5.43–21.12	5.76–32.50	4.09–23.51	24.85–95.94
Medium third									
Median	74.39^D^	13.03^AB^	59.46^CD^	31.67^ABCD^	18.89^ABC^	14.64^A^	49.32^ABCD^	14.83^A^	60.94
Min–Max	13.09–94.76	5.07–35.25	33.03–85.62	10.05–71.42	3.98–36.23	9.80–20.10	16.64–80.28	5.49–44.88	18.54–74.04

Superscript different letters indicate statistical differences among the groups

C+- positive control of contamination, without paste treatments

*Prop* propylene glycol, *DW* distilled water, *d* days, *U* ultrasonic agitation

The pastes effectiveness, at the superficial region of the dentinal mass in relation to the root canal, was worst for the G1 and G3 groups, but without statistical differences. G3 showed statistical differences only compared to G6 (CH + Prop + 15 + U) (*P* = 0.041). G1 did not present statistical differences with any other group, but, according to the median, there was a bacterial viability reduction of 22.7% compared to the control group, while the other groups (G2, G4, G5, G6, G7, and G8) obtained greater reductions of 41.5–84.7%. At the deep area of the dentinal tubules, G6 exerted the best antimicrobial capacity, followed by G8, G2, G5, G7, and G4.

### Microbiological culture evaluation

The calcium hydroxide pastes that remained for 7 days inside the root canals without ultrasonic agitation presented less antimicrobial efficiency for intratubular decontamination, with greater numbers of colony forming units per mL (CFU/mL) after the medications (G1 and G3). The CH paste with propylene glycol with ultrasonic agitation that remained for 15 days (G6) presented the lesser number of CFU/mL. After G6, the increasing order of CFU/mL was G7, G8, G2, and G5 (Table 2).

**Table 2:** Amount of CFU/mL per group for samples performed with Largo #5 (L5) and Largo #6 burs (L6), amount of total CFUs and amount of plates that did not present bacterial growth

Groups	G1	G2	G3	G4	G5	G6	G7	G8	C+
Largo #5	113^a,b^	13^a^	153^b^	45^a,b^	34^a^	0^a^	17^a^	13^a^	>100^b^
Largo #6	244^b^	17^a^	191^b^	69^a,b^	8^a^	7^a^	8^a^	13^a^	>100^b^
Total UFC/mL	357	30	344	114	42	7	25	26	>100
PWBG*	5	13	3	10	14	17	14	14	0

*Plates without bacterial growth, of a total of 20 plates in each group

^a,b^Different letters means statistical differences between columns

When comparing the decontamination at the superficial layer, i.e., at the excision of dentin with the #5 Largo bur, the CH paste with propylene glycol, ultrasonically agitated/15 days (G6) presented the best result, with zero CFU/mL. The groups that had the greater CFU/mL were the CH pastes for 7 days without ultrasonic agitation (G1 and G3). The comparisons were similar at the deep area of the dentinal tubules, by the dentinal excision with a #6 Largo bur.

In Table 2, it is possible to observe greater bacterial proliferation with the groups of 7 days of medication without ultrasonic agitation (G1 and G3), showing few plates without bacterial growth, similar to the positive control. G6 showed the smallest number of CFU/mL and the higher amount of plates without growth.

The Pearson correlation test was applied between these two methodologies—MC and CLSM analysis. The methods presented a positive correlation of medium level (correlation = 0.53) (*p* < 0.05), showing that these two evaluations produced similar results.

### Intratubular penetration evaluation

**Fig. 3:**
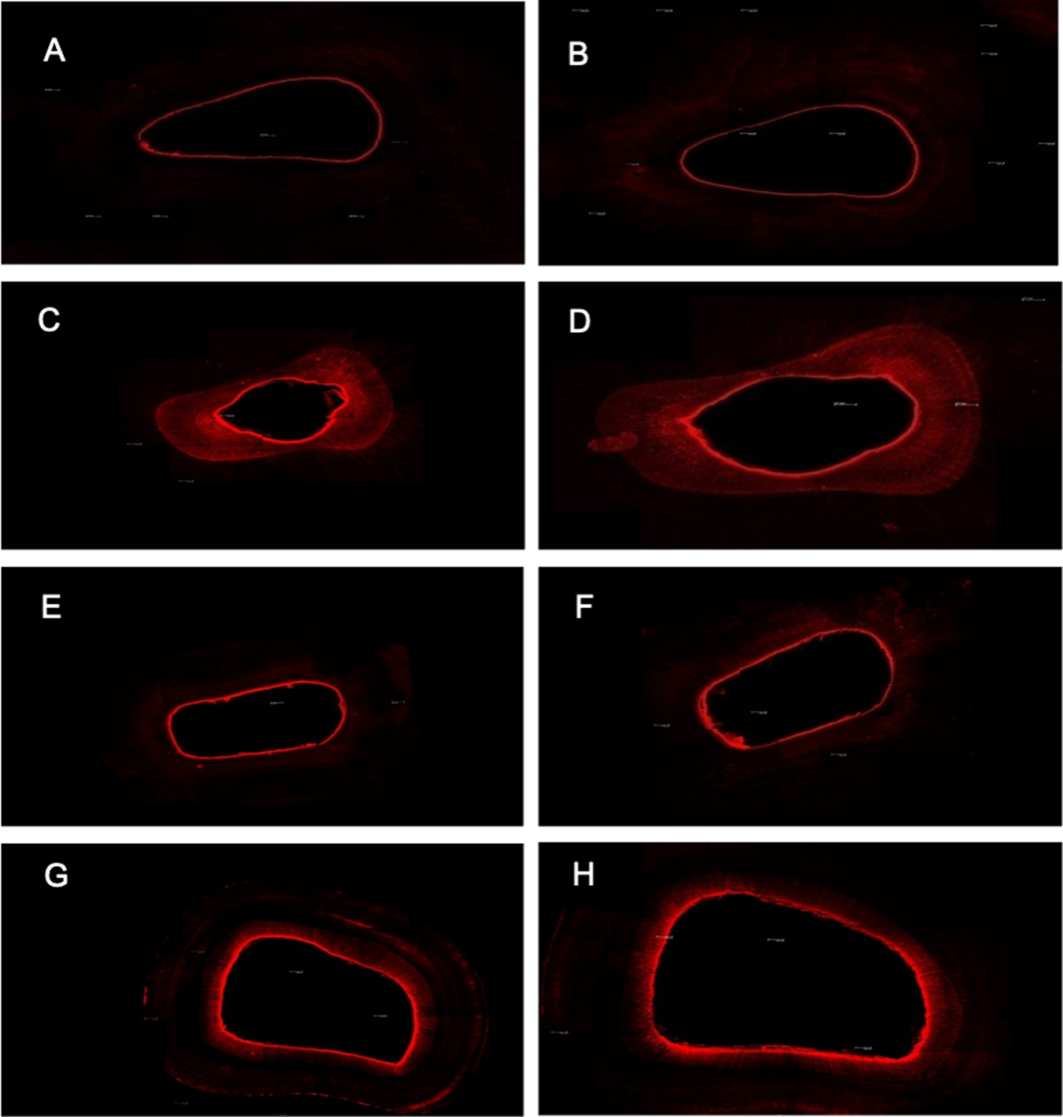
Images of the penetration of the calcium hydroxide pastes stained with 0.1% B Rodamine at the transversal cuts of 3 (left column) and 5 (right column) millimeters from the apical end of the specimen. **A** and **B** Calcium hydroxide with vehicle propylene glycol, (**C** and **D**) Calcium hydroxide with vehicle propylene glycol associated with the ultrasonic agitation, (**E** and **F**) Calcium hydroxide with vehicle distilled water, (**G and H**) Calcium hydroxide with vehicle distilled water associated with the ultrasonic agitation

The intratubular penetration of intracanal medications was evaluated at the surfaces of the transversal cuts at 3 and 5 millimeters from the apical border of the bovine dentin specimens. The ultrasonic agitation of the pastes promoted filling of the dentinal tubules in both vehicles. The best penetration was observed in the CH group with propylene glycol and ultrasonic agitation, showing statistical differences compared to the other groups (Fig. 2B). In both thirds, cervical and medium, the total penetration, considering the groups, was similar (Fig. 3, A-H).

## Discussion

Two methods were used to evaluate the antimicrobial effect of calcium hydroxide pastes submitted or not to ultrasonic agitation, MC and CLSM. Positive correlation obtained by the Pearson test showed reliable results from both methods. The MC does not evaluate the specimen as a whole, but by means of dentin debris, however, the challenge of bacteria to proliferate again, out of the teeth, is higher. The CLSM, on the other hand, evaluates the specimen in magnification, i.e., thin and small areas that can show three dimensions of images and where the bacteria are.

An experimental model to test medicaments of endodontic interest was described by Haapasalo & Orstarvik [25] utilizing bovine dentinal tubes and *E. faecalis* and has been largely used since then [26]. Inclusively, Camargo et al. [27] did not find statistical differences between human and bovine teeth, in relation to the diffusion of ions throughout the dentin specimen.

The present study was based on the bacterial viability by means of CLSM described by Andrade et al. [18] to contaminate all the specimens. The intention was to simulate in vitro the clinical situation of endodontic infections in the oral environment. Some important modifications of the anterior contamination methodologies were: dentine tubes of 12 mm; sealing the root external surfaces to obtain dentin contamination only through the canal; centrifugation protocol (adopted from Ma et al. [19]) of the specimens, but on alternate days to increase bacterial penetration; inocula concentration of 3 × 10^8^ CFU/mL after 7 h (bacteria growth curve of exponential proliferation); and 5 days of contamination with inocula renewal. Therefore, the confirmation of the best and more homogeneous intratubular bacterial proliferation was obtained. This modified methodology was recently utilized for testing dentin decontamination by different irrigants [28–30].

Although the viscous vehicles also are soluble in water, they promote slow ion liberation for prolonged times [7], a necessary issue in some clinical situations, such as the inhibition of inflammatory root resorption and periapical reactions repair [31]. It was observed in the present work, that the groups when propylene glycol was utilized, presented better results, mainly in the deep regions of the dentine, when compared to the distilled water groups, probably due to its greater facility to penetrate in tiny areas such as the tubules. This suggests that the smaller surface tension of propylene glycol contributes to the best diffusion of pastes inside the dentinal tubules, corroborating with Cruz et al. [9].

On topic Penetrability of the pastes by CLSM, we tried to simulate the clinical condition because of the penetration of the pastes was hard inside the tubules due to the superficial tension. In this experiment, with more time, corresponding to the clinical time of 7 days, the penetration was greater.

Through the Pearson correlation test, the obtained results of bacterial viability were inversely proportional to the data of the pastes penetration test, which means when there was proximity of the medications to the microorganisms, there was a greater elimination of them.

The ultrasonic agitation promoted better filling of the dentinal tubules, when compared to the same medication and period without agitation. This information can help increase the antimicrobial effect of the calcium hydroxide, thus, its action was enhanced due to the paste boosting by ultrasound, showing a better penetration, corroborating Duarte et al. [17] and Arias et al. [20].

Holland et al. [16] demonstrated the antimicrobial ineffectiveness of calcium hydroxide when maintained for 7 days only in the root canal. In the present work it was confirmed, with the G1 (CH + prop) and G3 (CH + distilled water) groups, the lowest effect of the pastes in a short period of time. However, when the same pastes were maintained for 7 days and ultrasonically agitated, there was better antimicrobial action, equivalent to the medications maintained for 15 days. These results were confirmed by both methodologies, CLSM and MC.

At the CLSM analysis, at the superficial as much as at the deep region, the G1 and G3 groups presented the least antimicrobial effectiveness. The best results in deep regions were reached by the G6 (CH + Prop/15d + U), G2 (CH + Prop/7d + U) and G8 (CH + dw/15d + U) groups, showing statistical differences compared to the positive control, confirming that ultrasonic agitation enhanced the antimicrobial action of medicaments in the deep areas of the dentine [17, 20].

Some studies reported dentin buffer capacity, such as the reduction of hydroxyl ions when a calcium hydroxide paste is introduced inside the root canal [32, 33]. However, with the present study, we can affirm that the difficult diffusion through the dentinal tubules is the main reason for the weak action of calcium hydroxide. When agitated with ultrasound, the pastes obtained better results [20]. The longer the medication remains inside the canals, the better will be its diffusion [34] and, with the use of ultrasonic agitation, this time was reduced. With the alkaline medication reaching the deep regions, resistant microorganisms can be affected, such as *E. faecalis*, which can survive until pH 11.5 [12]. Regarding the “buffer capacity” of the dentine, Freire et al. [35], utilized dentine powder mixed with calcium hydroxide. They observed an alkaline pH of this mix, i.e., the dentine did not exert the buffer activity in the mixture.

The importance of reducing the time of the inter-appointment medication, and obtaining a satisfactory decontamination, reduces the risk of coronary infiltration. Tennert et al. [36] showed that many fractures appear shortly after making a provisional coronary sealing in the materials. In addition to coronary infiltration, as soon as the endodontic treatment is completed, the patient rehabilitation will improve, and the risk of teeth fracture will decrease.

Therefore, the pastes maintained for 15 days and maintained for 7 days with ultrasonic agitation (G2, G4, G5, G6, G7, and G8) promoted the best antimicrobial action in both tested vehicles. In addition, in the deep regions, medications with propylene glycol were more effective. According to these results, we recommended the introduction of ultrasonic agitation of calcium hydroxide pastes in the clinical routine, reducing the time of the intracanal medication from 15 to 7 days, aiming for better decontamination and optimizing the endodontic treatment.

## Conclusion

All pastes demonstrated better penetration and antimicrobial activity against *E. faecalis* with both vehicles when agitated with ultrasound, even in periods of 7 days. The vehicle propylene glycol showed better antimicrobial results and penetration, mainly under agitation. Therefore, the optimization of the endodontic treatment can be achieved by ultrasonic agitation of the intracanal dressing, remaining only 7 days inside the root canals between sessions.
